# 

**Published:** 1887-08

**Authors:** 


### CORRESPONDENCE.

### International Congress—General Programme of the Congress.

As there appears to be a very general desire, both at home and abroad, to have the programme of arrangement for the meeting of the International Medical Congress made public, I herewith submit the formula therefor determined upon by the Committee of Arrangements intrusted with that duty.

FIRST DAY—MONDAY, SEPTEMBER 5.

The Congress will assemble at Albaugh's Opera House at 11 A. M., and will be formally opened by the President of the United States, to be followed by a short address of welcome by the Secretary of State; Address by the President of the Congress; Report of Secretary-General and Chairman of Committee of Arrangements. Adjourn at 1:30 P. M. From 3 to 6 P. M., meeting of the Sections at their respective halls. Evening *conversazione* at U. S. Pension Hall from 8 to 11 P. M.

SECOND DAY—TUESDAY, SEPTEMBER 6.

Meeting at 10 A. M. at Albaugh's Opera House. General Addresses by Drs. Flint and Semmola. Sections will meet at 11 A. M., and adjourn at the same hour with Congress at 1 P. M. In the afternoon the Sections will meet from 3 to 6 P. M. In the evening it is expected that a reception will be given by the President of the United States, and the Corcoran Art Gallery will be thrown open to the members and their families.

THIRD DAY--WEDNESDAY, SEPTEMBER 7.

The Congress will meet at 10 A. M. General Addresses until 1 P. M. The Sections will meet as usual at 11 A. M., and adjourn at 1 P. M. Afternoon meeting of the Sections from 3 to 6 P. M. Evening reception to the members and their families by the citizens of Washington.

FOURTH DAY--THURSDAY, 8TH.

General meeting at 10 A. M. Addresses, if not previously delivered. Meeting of the Sections at 11 A. M.; adjourn at 1 P. M. Afternoon, Sections meet from 3 to 6 P. M. General reception buffet banquet at U. S. Pension Hall from 8 to 11 P. M.

FIFTH DAY--FRIDAY, 9TH.

General meeting at 10 A. M. Transaction of business affairs of Congress. Meeting of Sections at 11, and adjourn at 1 P. M. Afternoon, Sections meet from 3 to 6 P. M.

SIXTH DAY—SATURDAY, 10TH.

General meeting at 10 A. M. Adjourn at 11 for visit to Mt. Vernon.

On Sunday or Monday, the day not yet determined upon, an excursion train will leave Washington with the foreign members and their families for Niagara Falls, under the escort of a part of the Committee of Arrangements, selecting the route which will afford our foreign brethren an opportunity to see some of the most interesting and thrifty portions of our country, as well as very beautiful scenery.

In completing the details of this programme it may be necessary to make some slight modifications.

I send herewith an important communication from the Chairman of the Sub-Committee on Transportation, Dr. J. W. H. Lovejoy.

ALEX. Y. P. GARNETT, M. D.,
*Chairman of Committee of Arrangements.*

RAILWAY RATES TO WASHINGTON.--The Railroad Associations which have already agreed to make a reduction of fare for members of the Congress and their families on the roads under their control are:

The Trunk Line Association.

The Central Traffic Association.

The Newport News and Mississippi Valley Company.

The Southern Passenger Association.

These cover the greater part of the territory east of the Missouri and Mississippi rivers. The whole list of roads controlled by these Associations is too large for publication, but members can obtain all the necessary information by application to the railroad agent at the starting point. It will be required to pay full fare to Washington, and a return will be allowed for "one-third the highest limited fare" on the Association's certificate. It will be necessary for these certificates to be procured before starting and have upon them the receipt of the railroad agent for the full fare to Washington. Members intending to attend the Congress should, as soon as possible, make application to the undersigned for blank certificates of the Association over whose roads they intend to travel, and the blanks will be forwarded at as early a date as they can be obtained. A separate certificate will be required for each person.

J. W. H. Lovejoy, M. D.,
*Chairman Transportation Committee.*

No. 900 12th St., Washington, D. C.

### Duty on Exhibits for the Congress.

[The following letter from the Secretary of the Treasury to Dr. John B. Hamilton, Secretary-General of the Ninth International Medical Congress, is of especial interest to our foreign confreres who wish to attend the Congress:]

*Sir:* In reply to the inquiries contained in the letter of F. H. Rehwinkel, M. D., referred by you to this department, I have to state that the professional books, implements, instruments, . . . of persons arriving in the United States are exempt from duty under the provisions of the tariff law, which also provides for the free admission of "models of inventions and other improvements in the arts, such as cannot be fitted for use," and that parties arriving from foreign countries for the purpose of attending the

Ninth International Medical Congress, to meet at Washington, September 5, 1887, and who bring with them their own "surgical or dental instruments, scientific and mechanical appliances, models and materials to be used for clinical demonstration under the direction of said Congress," will be entitled to have the same passed free of duty, on the usual oath that they are for their personal use, and are not intended for sale.

As it would appear from the act of Congress relating to said convention that the said Ninth International Medical Congress is a society established for philosophical and scientific purposes—books, maps, and charts (not more than two copies in any one invoice), philosophical and scientific apparatus, instruments, and preparations, casts, paintings, drawings and etchings, specially imported in good faith for the use of said Congress and not intended for sale, would also seem to be entitled to free entry under the provisions of the tariff act now in force, a copy of which is herewith enclosed. (See paragraphs 660, 759, and 815.)

Copies of this letter will be transmitted to the Collectors at the principal ports for their information and guidance.

Respectfully yours,

(Signed) C. S. FAIRCHILD,
*Secretary of the Treasury.*

### EDUCATIONAL.

### University of Tennessee—Dental Department.

The meeting of the Southern Dental Association at the Hygeia Hotel, Old Point Comfort, Va., August 30, promises to be a grand success, if not the largest meeting of the Dental profession on the continent. The Central Traffic Association as well as the Southern Passenger Association will give reduced rates to those who wish to attend. Those purchasing tickets from the Central Traffic Association agents will get certificates from railroad agent when procuring tickets, which will enable the holder to return from "Old Point" for one third fare.

Those who wish to take advantage of the reduction offered by the Southern Passenger Association will apply to Dr. J. Y. Crawford, Corresponding Seeretary, Nashville, Tenn., for certificates which will enable the holder to make the trip by paying one and one-third fare, tickets to be good twenty-four hours after adjournment of meeting.

The Virginians and our noble grand old President, Dr. W. W. H. Thackston, are exerting themselves to make the meeting a grand one. A cordial invitation is extended to all, East, West, North, and South.

J. Y. Crawford, Corresponding Secretary.

### EDITORIAL.

### Ninth International Medical Congress, R. R. Rates.

Let none of our readers, in any way interested in the International Medical Congress of September 5, 1887, omit reading carefully the official announcement concerning railroad rates over the signature of the Chairman of the Transportation Committee on page 415 of this number. They will see that the reduced rates apply not only to *members*, but also to members of *their families*. We wish to emphasize the fact, however, that all who wish to avail themselves of the reduced rates *must* apply to J. W. H. Lovejoy, M.D., No. 900 Twelfth street, Washington, D. C., for just as many *certificates* as they have *persons* intending to go. Such applications had better be made without delay.

### Registration Blank.

"The Congress will consist of such members of the regular medical profession as shall have registered and taken out their tickets of admission, and of such other scientific men as the Executive Committee of the Congress shall deem it desirable to admit." The fee has been fixed at ten dollars. Each member of the Congress will be entitled to a copy of the transactions.—*Rules of the Executive Committee.*

Those about to register will please read and give attention to the following directions to secure accuracy in the list of members of the Congress; and, to prevent mistakes therein, it is especially requested that all proper names of persons and places shall be written distinctly and in full, and without abbreviations in any case. (*For example:* Instead of J. W. Taylor, Phil., Pa., it should be written John Warren Taylor, Philadelphia, Pennsylvania, etc., etc.)

By order of COMMITTEE OF ARRANGEMENTS.

*Register No*...............

FORM TO BE FILLED UP BY APPLICANT FOR MEMBERSHIP IN THE CONGRESS.

*Data required:*

Name in full.................................................................

Post Office address..........................................................

If in a city, give also street and number....................................

If in the country, give also name of the county..............................

State.....................................................................

Province..................................................................

County....................................................................

If a Delegate, state from what county or society.............................

Practice general or special..................................................

If special, name the branch .................................................

CONCERNING A COMMEMORATIVE MEDAL.

As former International Medical Congresses have been commemorated by suitable medals, it is proposed (should a sufficient number be subscribed for) to have one of good size and proper design struck for the IX Congress. A medal with the head of George Washington on the one side and the seal adopted for the Congress on the other, would doubtless be appropriate. The medal of the London Congress had the bust of the Queen on one side and the seal of the Congress on the obverse, and cost one guinea. Such a medal, well executed in bronze, will cost us five dollars. Those approving the project and desiring such a medal will deposit five dollars with the treasurer, who will give a receipt for the same, and deliver the medal in proper time, if struck.

Blank form to be filled:

Name in full..............................................................

Post Office address.......................................................

Number of medals subscribed for...........................................

If from any cause the medal should not be struck, the money will be returned to subscribers.

### CONTENTS.

COMMUNICATIONS.

**A MONTHLY MAGAZINE DEVOTED TO THE INTERESTS OF THE**

### DENTAL PROFESSION.

*Terms Reduced to* $2.00 *Per Year. Single Copies,* 25 *Cents.*

**EDITED BY PROF. J. TAFT, D.D.S.**

—AND—

**Published by SAM'L A. CROCKER & CO., Ohio Dental and Surgical Depot.**

The volume commences in January and closes in December.

The REGISTER being issued monthly is always fresh, therefore Indispensable to the practitioner who desires to keep posted up with the new inventions and improvements which are daily developed. Neither expense nor effort will be spared to give value and variety to its contents. Articles will be illustrated with well executed engravings whenever they will add to the interest or assist to explanation.

Its extensive circulation and frequency of issue make it one of the BEST DENTAL ADVERTISING MEDIUMS in the United States.

All subscriptions must begin with January or July.

Local or special notices, five lines or less, will be inserted for $3 each insertion; over five lines, 50 cts. per line.

All advertising bills payable in advance, or at furthest, after the issue of the first number containing the advertisement. All transient advertisements to be paid for in advance.

☞CONTRIBUTIONS SOLICITED.

Exclusively Editorial Communications should be addressed to the Editor.

The latest number of the REGISTER may be ordered with or without other goods at any time, and all letters should be addressed to

**SAM'L A. CROCKER & CO., Ohio Dental and Surgical Depot, Cincinnati, O.**

